# Health care equity and access for marginalised young people: a longitudinal qualitative study exploring health system navigation in Australia

**DOI:** 10.1186/s12939-019-0941-2

**Published:** 2019-03-04

**Authors:** Fiona Robards, Melissa Kang, Katharine Steinbeck, Catherine Hawke, Stephen Jan, Lena Sanci, Ying Ying Liew, Marlene Kong, Tim Usherwood

**Affiliations:** 1 Department of General Practice, Westmead Clinical School, The University of Sydney, PO Box 154, Westmead, NSW 2145 Australia; 2 University of Technology Sydney, Discipline of Public Health, Sydney, Australia; 3 The University of Sydney, Discipline of Paediatrics and Adolescent Health, Sydney, Australia; 4 The University of Sydney, School of Rural Health, Orange, Australia; 5 The George Institute for Global Health, University of New South Wales, Sydney, Australia; 6 Department of General Practice, University of Melbourne, Carlton, Australia; 7 The Kirby Institute, University of New South Wales, Randwick, Australia

**Keywords:** Young people, Adolescents, Access to health care, Health system navigation, Marginalised youth, Technologies

## Abstract

**Background:**

Young people have unique social, emotional and developmental needs that require a welcoming and responsive health system, and policies that support their access to health care. Those who are socially or culturally marginalised may face additional challenges in navigating health care, contributing to health inequity. The aim of this study was to understand health system navigation, including the role of technology, for young people belonging to one or more marginalised groups, in order to inform youth health policy in New South Wales, Australia.

**Methods:**

This qualitative longitudinal study involved 2–4 interviews each over 6 to 12 months with marginalised young people aged 12–24 years living in NSW. The analysis used Nvivo software and grounded theory.

**Results:**

We interviewed 41 young people at baseline who were living in rural or remote areas, sexuality and/or gender diverse, refugee, homeless, and/or Aboriginal. A retention rate of over 85% was achieved. Nineteen belonged to more than one marginalised group allowing an exploration of intersectionality. General practitioners (family physicians) were the most commonly accessed service throughout the study period.

Participants were ambivalent about their healthcare journeys. Qualitative analysis identified five themes:

1. Technology brings opportunities to understand, connect and engage with services
2. Healthcare journeys are shaped by decisions weighing up convenience, engagement, effectiveness and affordability.
3. Marginalised young people perceive and experience multiple forms of discrimination leading to forgone care.
4. Multiple marginalisation makes health system navigation more challenging
5. The impact of health system complexity and fragmentation may be mitigated by system knowledge and navigation support

**Conclusions:**

The compounding effects of multiple discrimination and access barriers were experienced more strongly for young people belonging to mutiple marginalised groups. We identify several areas for improving clinical practice and policy. Integrating technology and social media into processes that facilitate access and navigation, providing respectful and welcoming services that recognise diversity, improving health literacy and involving professionals in advocacy and navigation support may help to address these issues.

**Electronic supplementary material:**

The online version of this article (10.1186/s12939-019-0941-2) contains supplementary material, which is available to authorized users.

## Background

Achieving health equity requires ensuring *universal healthcare access* for all young people, including those most disadvantaged. Young people who are marginalised due to their socio-demographic background or for other reasons may face barriers to accessing health care [1].

To date exploration of young people’s access to health services has mainly involved cross-sectional studies that identify and quantify barriers and enablers to access [1, 2]. Models of care have evolved from static conceptions of youth-friendly services, rather than movement around the health system [3]. Since health needs might require a range of services over time, exploring navigation would add a further dimension to our understanding of access.

A systematic review of the international literature on access and navigation of health care by marginalised young people found that most studies have focused on single marginalised groups with little exploration of the impact of young people who experience multiple disadvantage [4]. Access barriers are exacerbated for groups of marginalised young people. For example, cost particularly affects low income and homeless young people, confidentially concerns and service location were more prominent for young people living in rural areas, discrimination affects gender and sexuality and gender diverse young people, and cultural issues were salient for refugee and Indigenous young people [4]. Achieving health equity involves promoting access across a range of marginalised groups, including those facing multiple disadvantage.

There has also been limited exploration of the role of technology in healthcare access, although given its ubiquity in young people’s lives it is likely to play a part in help-seeking. Current evidence is inconclusive about the effectiveness of online services to promote access [5], but some studies with marginalised young people suggest there is promise for technology to enhance communication with young clients who utilise face-to-face services [6, 7].

Research in Australia prior to widespread use of smartphones to access the internet identified barriers to access for young people as including concerns about confidentiality, structural factors such as cost and transport, and lack of awareness of the range of services available [8]. Studies focusing on single marginalised groups of young people in Australia found similar barriers, but noted that these could be more prominent among different populations. For example, in rural areas, structural barriers such as service availability, transport and cost as well as personal barriers such as confidentiality concerns and stigma could be particularly salient, while among Aboriginal young people shame and stigma were important [4].

The Australian health system has been described as several different but connected systems, with funding and policy from federal, state and local governments as well as the private sector [9]. Although Australia has a universal health insurance scheme (“Medicare”) to enable access to a wide range of health services at reduced or no cost, there is evidence that out of pocket costs are increasing [10] and that health care has become more fragmented [11, 12]. General Practitioners (GPs) deliver primary health care and provide referrals to other specialists and are therefore the ‘gatekeepers’ to much of the health system in Australia.

This longitudinal study explored young people’s journeys through the health system in New South Wales (NSW), Australia, over time. The aim was to understand health system navigation, including the use of technology, for young people belonging to one or more marginalised groups.

## Methods

This was one of four discrete but interrelated studies that formed a mixed-methods study series known as the *Access 3* project in NSW, the most populous state in Australia, and which has approximately 1.27 million people aged 12–24 years’ [13]. The *Access 3* project explored barriers to health care and health system navigation, and aimed to inform policy. The four studies included: 1. a cross-sectional survey to describe and quantify barriers, facilitators, and how technology is used, to access healthcare; 2. a longitudinal, qualitative study focusing on marginalised young people, to explore their journeys over time through the health system; 3. A qualitative cross-sectional study to obtain the perspectives of health professionals; and 4. A policy translation forum to translate synthesised data into policy-ready recommendations via a facilitated workshop with stakeholders. We have published a detailed description of methods [14].

### Design

This longitudinal qualitative study (Study 2 in the *Access 3* project), used a series of one-on-one semi-structured interviews over 12 months with marginalised young people. A group of Youth Consultants, (young people from marginalised backgrounds employed to advise about the study) provided advice via email and group meetings on the survey instrument, recruitment methods, interview questions, interpretation of findings, policy translation and dissemination.

### Participants

Young people (12–24 years) belonging to one or more of the following groups were recruited: living in rural or remote regions, homeless, of refugee background, Aboriginal, and/or sexuality and/or gender diverse. To be eligible, they also had to have had contact with the health system in the previous six months for a chronic or complex health condition and/or disability, so it was likely they would have a need to access health care across the study period.

### Recruitment

Participants were a sub-sample of respondents to a cross-sectional survey, the first study of the *Access 3* series which ran for twelve months from February 2017. The survey oversampled young people from each of the five target marginalised groups. At the end of the survey all participants were asked if they would like to be part of a second study that would involve four interviews over 12 months, in person or by phone/Skype.

The survey participants who indicated an interest to be part of the second study, were purposively selected based on answers to identifier questions and invited to participate in the second study [14]. A range of specific questions was included as identifiers for our five target groups of marginalised young people. These are summarised in Table 1.

**Table 1:** Summary of identifier questions for marginalisation

Marginalised group	Identifier questions in survey
Aboriginal and/ or Torres Strait Islander	Based on response to “Are you Aboriginal and/ or Torres Strait Islander?”
Homeless	Based on response to ‘current living situation’ (15 options including ‘Other’).
Refugee	If born overseas and moved to Australia as refugee or asylum seeker.
Rural/ remote	Based on postcode of residence and Australian Standard Geographic Classification of Remoteness.
Sexuality and/ or gender diverse	Based on selection of one or more of a range of responses to questions about sexual attraction, sexual identity, gender identity and having an intersex condition.

The widely accepted ‘cultural definition’ of homelessness was used - including primary (without conventional accommodation), secondary (moving between various forms of temporary shelter) and tertiary levels of homelessness (people living in accommodation without adequate facilities and security of tenure) [15]. Additional identifier questions asked participants whether there had been contact with health system within past 6 months that involved hospital admission, presentation to an emergency department or because of a chronic or complex health condition.

The survey measured health status using the Kessler-10 (K10) questionnaire [16, 17] and the World Health Organisation Wellbeing Index (WHO-5) [18]. The K10 is a validated instrument that provides a measure of non-specific psychological distress in adolescents and adults, relating to symptoms of anxiety and depression experienced in the most recent four-week period. The 10-item questionnaire is scored, with total score being classified into ‘low’, ‘moderate’, ‘high’ and ‘very high’ levels of psychological distress. The WHO-5 is a 5-item validated questionnaire that measures wellbeing [18]. Each of the 5 items is scored from 5 (all of the time) to 0 (none of the time). The raw scores can range from 0 (absence of well-being) to 25 (maximum well-being) and are converted into a percentage. Participants were also asked to rate their health (‘excellent’, ‘very good’, ‘good’, ‘fair’ or ‘poor’) and select chronic mental and physical health conditions from a predefined list.

Potential participants were approached by email with information about a second study, including that the purpose of the study was to inform the development of youth health policy. This was followed up with a more informal email and SMS. Researchers were introduced as employees of the University of Sydney Participants were not previously known to the researchers. Of the 106 young people contacted, 16 declined to participate and 49 did not respond. The remainder were recruited.

### Data collection

We conducted two, three or four interviews with each participant over 6–12 months. The number of interviews varied due to late recruitment of some participants, and some interviews being missed. These were face-to-face, by telephone or Skype, and audio-recorded and transcribed. One participant declined audio recording. Handwritten notes were taken in all interviews. Interviews were conducted by two female authors (FR, a Senior Research Officer and PhD candidate; and YYL, trainee General Practitioner. FR had postgraduate level training in qualitative research and both FR and YYL have interest in adolescent health). Interviews lasted between 30 min and two hours. Interviews were held in quiet and confidential spaces such as libraries, health services, youth services and university buildings. One participant aged under 14 years chose to have his mother present at the first two of four interviews. Two refugee participants chose to use telephone interpreters. Participants received a shopping voucher of 30 Australian dollars for each of the first three interviews and 50 dollars for the final interview. The interview schedule included questions about the types of contact with health services during the interval between each interview, access and barriers to health services, factors influencing decisions about help-seeking, follow-up and referral plans, and the perceived quality of health care received. This allowed for an exploration of navigation through the health system over time. The interview questions were pilot tested with five of the study youth consultants and adjustments made. The interview guide was shaped by the study aims, however the questions were revised across interviews so that the researchers could explore in-depth new themes that were emerging but unclear. Examples of the interview questions are provided in a supplementary file [see Additional file 1 sample interview questions]. The ‘health system’ was defined broadly as any service delivering healthcare, including online services. Of the six participants who withdrew from the study five contacted the researchers via email citing reasons of time and other commitments such as school, work and travel.

### Data analysis

Sociodemographic characteristics, self-reported health status, psychological distress as measured by the Kessler K10 scale [16], and the presence of chronic health conditions were extracted from the survey data for the participants in the current study. At each interview over the study period, information about number and type of health services accessed since the previous interview was obtained and quantified.

Interview transcripts were reviewed for accuracy by researchers (not participants) and entered into NVivo 11 [19]. To understand health system navigation over time, the combined set of transcripts from each participant were also examined together, and in order. Three authors undertook the data coding and analysis (FR, YYL and MK) and followed the method outlined by Corbin and Strauss [20]. This method involved making data comparisons (where line by line data from each transcript are compared for similarities and differences). In addition, questions were asked of the data to make connections between concepts, for example: ‘How do participant’s experiences vary across the health system?’; ‘How do participants go about deciding which health service to access?’; ‘Were there changes over time and, if so, what influenced these?’; ‘Were themes common among all groups or experienced differently?’; ‘What facilitated health system navigation?’ and ‘What contributed to forgone care?’. These processes initially enabled coding of the more fine-grained, lower-level concepts which, in our data, were the factors influencing how marginalised young people navigate the healthcare system. Higher-level themes were then developed by looking across the lower-level concepts and considering how they fit together (using NVivo sets to group codes, examining memos written about emerging themes during the data collection and analysis process, and through team discussion of themes). Corbin and Strauss [20] describe the coding levels as elements of an umbrella where the lower-level concepts are the spokes and the higher-level themes are like the cloth that covers the spokes to make the umbrella work as a whole. Our aim was to arrive at a workable conceptual framework (our ‘umbrella’) for the factors influencing marginalised young people’s navigation of the health system. The higher-level analysis also considered the longitudinal nature of the data. Data and theoretical saturation was reached, meaning that no new concepts or themes were emerging [21].

## Results

The interviews were conducted from March 2016 to May 2017. Forty-one young people participated in baseline interviews, five withdrew after Interview 1 and one after Interview 2, citing time and commitment constraints, giving a retention rate of over 85%. Four of the six who withdrew belonged to only one marginalised group. Twenty-eight participants (68.3%) completed four interviews, three (7.3%) participated in three interviews, five (12.2%) had two interviews and five had one interview (12.2%). Most interviews were spaced between 3 and 4 months apart over 12 months, however due to late recruitment, four participants completed two or three interviews over six months. All interview data were included in analysis.

The mean age of the sample was 19.3 years (range 12–24 years). There were 21 rural participants, 20 sexuality and/or gender diverse (including seven gender diverse and one intersex), nine homeless, nine refugee, and five Aboriginal participants. Nineteen participants (46%) belonged to more than one marginalised group: 15 belonged to two groups (37%), and four belonged to three groups (10%)(Tables 2 and 3).

**Table 2:** Intersections between marginalised groups (*n* = 41)

Belong to 1 marginalised group	n	Belong to 2 marginalised groups	n	Belong to 3 marginalised groups	n
Rural	6	Sexuality and/or gender diverse, rural	5	Sexuality and/or gender diverse, rural, homeless	3
Sexuality and/or gender diverse	9	Sexuality and/or gender diverse, homeless	3	Aboriginal, homeless, rural	1
Homeless	1	Aboriginal, rural	3		
Aboriginal	1	Refugee, rural	3		
Refugee	5	Refugee, homeless	1		
Total	22	Total	15	Total	4

**Table 3:** Sociodemographic and sociocultural characteristics of baseline sample (*n* = 41)

	N (%)
Gender	
Female (two gender diverse)	30 (73.2)
Male (two gender diverse)	8 (19.5)
Other (all gender diverse)	3 (7.3%)
Education	
High school	12 (29.3)
Intensive English Centre (IEC) high school	2 (4.9)
Full-time tertiary studies	15 (36.6)
Part-time tertiary studies	2 (4.9)
Not studying at all	9 (22.0)
Current employment	
In full-time paid work	4 (9.8)
In part-time or casual work	16 (39.0)
Unemployed, looking for work	8 (19.5)
Unemployed, not looking for work, studying	8 (19.5)
Unable to work due to sickness or disability	3 (7.3)
A carer or doing home duties full time or part time	1 (2.4)
Other (volunteer work)	1 (2.4)
Internet and mobile access	
Internet access	37 (90.2)
Other internet access	3 (7.3)
No internet access	1 (2.4)
Youth allowance, Medicare care, Healthcare card, Private Health Insurance	
Own Medicare card^a^	28 (68.3)
Healthcare card^b^	20 (48.8)
Youth allowance^c^	15 (36.6)

^a^A government issued card that enables access to a range of medical services and prescriptions at a lower cost, and free care as a public patient in a public hospital. Young people are eligible to get their own card that is separate from their family card from 15 years of age

^b^A concession card issued by the government to enable access to subsidised medicines

^c^Government financial assistance for young people aged 24 or younger who are studying, doing an apprenticeship, looking for work or who have a health condition

### Health status

28/41 (68%) of participants rated their health as ‘excellent’, ‘very good’ or ‘good’, 29/39 (74%) had high or very high levels of mental distress as measured by the K10, 11/37 (29.7%) had low mood and 14/37 (37.8%) had likely depression as measured by the WHO-5, and 26/41 (61%) reported having a chronic physical or mental health issue (Table 4).

**Table 4:** Respondents’ health characteristics

Self-reported health rating (Total sample *n* = 41)	n (%)
Poor	6 (14.6)
Fair	7 (17.1)
Good	14 (34.1)
Very good	8 (19.5)
Excellent	6 (14.6)
Level of psychological distress (K10 score) (*n* = 39) (range 12–46/50, average 29.4)	
Low (10–15)	2 (5.1)
Moderate (16–21)	8 (20.5)
High (22–29)	8 (20.5)
Very high (30–50)	21 (53.8)
Wellbeing (WHO-5 score) (*n* = 37)	
Good wellbeing (51–100)	12 (32.4)
Low mood (29–50)	11 (29.7)
Likely depression (0–28)	14 (37.8)
Chronic health conditions (n = 41)	
Mental illness and/or drug and alcohol problem	22 (53.7)
Chronic physical health condition	10 (24.4)
Disability	3 (7.3)
None of the above	15 (36.6)

### Health care access

Table 5 shows the number and breadth of services and health professional types accessed by participants over 18 months. Data collected in the baseline interview (Wave 1) reflects service types accessed over the previous six months. Subsequent interviews captured services visited over periods of 3 to 4 months (Waves 2–4). Services visited included primary, secondary and tertiary care, including hospitals, community-based services and online care. GPs were the most frequently used service, being accessed by all participants at Wave 1 interviews and approximately two-thirds of participants at Interviews in Waves 2–4. Pharmacists and counsellors were the next most visited service types.

**Table 5:** Service and health professional types accessed over 18 months

Professional/service type^a^	Wave 1	Wave 2	Wave 3	Wave 4
Number of interviews in wave^b^	*n* = 41(%)	*n* = 34 (%)	*n* = 31(%)	*n* = 30(%)
General Practitioner^**c**^	41(100.0)	23(67.6)	24(77.4)	20(66.7)
Pharmacy	19(46.3)	7(20.6)	11(35.5)	10(33.3)
Counsellor	13(31.7)	5(14.7)	9(29.0)	11(36.7)
Dentist	14(34.1)	4(11.8)	8(25.8)	8(26.7)
Specialist	13(31.7)	0(0.0)	12(38.7)	6(20.0)
Hospital	14(34.1)	5(14.7)	1(3.2)	3(10.0)
Emergency Department	10(24.4)	5(14.7)	3(9.7)	4(13.3)
Pathology/medical imaging	3(7.3)	4(11.8)	2(6.5)	8(26.7)
Non-Government Organisation support	3(7.3)	3(8.8)	6(19.4)	5(16.7)
headspace	9(22.0)	1(2.9)	2(6.5)	3(10.0)
Nurse	4(9.8)	2(5.9)	4(12.9)	4(13.3)
Psychiatry	5(12.2)	2(5.9)	2(6.5)	4(13.3)
Online support	9(22.0)	2(5.9)	1(3.2)	1(3.3)
Optometrist	5(12.2)	4(11.8)	1(3.2)	3(10.0)
Sexual Health Service	5(12.2)	1(2.9)	3(9.7)	2(6.7)
Physiotherapist	5(12.2)	0(0.0)	0(0.0)	3(10.0)
Mental Health Service	5(12.2)	1(2.9)	1(3.2)	0(0.0)
Ambulance	1(2.4)	3(8.8)	1(3.2)	1(3.3)
Aboriginal Medical Service	2(4.9)	1(2.9)	1(3.2)	1(3.3)
Other health service^**d**^	9(22.0)	3(8.8)	3(9.7)	4(13.3)
none	0(0.0)	8(23.5)	2(6.5)	2(6.7)

^a^Not occasions of service

^b^A wave is the time period in which the interview occurred within the study period. Not all participants completed interviews in each wave

^c^Family Physician

^d^Included youth and adult cancer services, occupational therapy, chiropractor, pregnancy termination service, podiatrist, school first aid, mother’s support group

### Themes

**Fig. 1:**
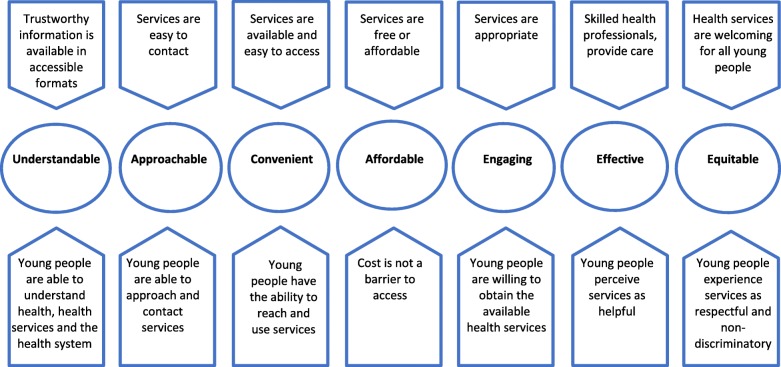
Factors influencing how marginalised young people navigate health system

As a result of the initial coding of interviews, lower-level concepts describing the factors that influence how marginalised young people navigate the health system, were elicited (circled in Fig. 1). These factors are both contextual pentagons above the circles) and relate to the characteristics of young people (pentagons below the circles) (Fig. 1).

Although the factors influencing how marginalised young people navigate health system were distinct, with in-depth analysis of data, the overarching theme ‘ambivalent journeys’ was found to describe participants’ often uncertain movements through a complex, fragmented system over time. Although participants matured over the study period, many experienced ongoing uncertainty and persistent forgone care as they alternatively embraced and resisted engaging in healthcare. Five common themes emerged from the higher-level analysis, described below noting where the theme was experienced differently, or more frequently, by specific marginalised groups.

### Theme 1: Technology brings opportunities to understand, connect and engage with services

The researchers observed many participants had a limited understanding of health, a health service’s role and availability, and how to access services, and this did not improve substantially over the duration of the study. However, they also described using technologies to help them make decisions about accessing healthcare.

*I did a bit of Googling and so I thought that it just wasn’t severe enough to go. But if it happens again, I’ll definitely book an appointment.* -Male, 18 years, homeless

Participants living in rural and remote areas said they were aware that they probably had a limited understanding of the possibilities of what services could be available in urban areas – a lack of service availability, particularly in rural areas, led to health issues not being addressed across the study period.

*Growing up I’ve been oblivious to accessing health services, always relying on my parents just to take me to the doctor. But then, sort of my maturing, and becoming more aware... I’ve realised how limited… the sources, and all that sort of stuff. How it can be a bit to our disadvantage when trying to access health services, like travelling to [regional town] or [major city]– and just the cost involved with that, the time, the effort. So, it can be draining, and it can make some people not seek that further medical assistance.*-Female, 17 years, remote

Participants’ skills in locating information varied - having information that was relevant, current, trustworthy and understandable was valued. They trusted government and organisational websites and valued individual service details about approach in order to assess fit. For example, sexuality and gender diverse young people valued online information to locate professionals who identified as allies, resulting in better ongoing engagement.

*Making it clear that you are understood, that there’s gonna be some information for you, it’s probably the biggest one. Or even if [they] had something on their website, just say that they were you know queer and trans-friendly... that would make me comfortable... so you get an indication from the get-go that you’re picking the right service for you because I’ve found that’s really hard.* -Female, 23 years, sexuality diverse, homeless

Participants suggested service promotion via social media would make finding information easier. Online health and service information empowered young people, but it also led to expectations about treatment and professionals’ expertise which was not always met in practice. Technology was integrated into information and help-seeking throughout the study period.

When asked for ways to make the health system easier to navigate, even those who had not previously used online services and who valued face-to-face connections, overwhelmingly suggested technology-based solutions. Participants wanted services to be technology-enabled to enhance convenience. Technology solutions included the ability to contact services, access to Wi-Fi, use of clinical tools in and between sessions, online documentation and navigation support. For example, participants valued being able to contact and book a service online.

*I like when booking and that kind of stuff is online... talking on the phone’s very alien to me... I prefer not to. So, I like to be able to manage appointments and all that kind of stuff online.* -Female, 23 years, sexuality diverse, homeless

Apps helped monitor chronic conditions (such as diabetes). Navigation support included text reminders as part of active follow-up and pharmacy apps that provide reminders and help manage documentation.

*There’s an app that’s through the pharmacy… it tells me when I need my new scripts, or how long until my new script ends, if I have any repeats. That’s been really good. I use that tool a lot.*-Female, 24 years, Aboriginal, rural

Online services such as mental health counselling provided via online chat, were significant for some young people accessing care in a crisis, (usually after hours), and were a stepping stone to connection with an ongoing face-to-face service.

*I usually use, online services, if… I’m having trouble in that moment. If I just need some advice to get me through what’s happening right now, and face-to-face is sort of longer-term multiple appointment.*-Male, 19 years, sexuality and gender diverse

*Talking to someone online or at least approaching the issue online for the first time has meant that I’ve been able to kind of move past some of that anxiety and then maybe engage with face-to-face services and get more help… just diving into face-to-face… can be a bit intimidating. -*Female, 23 years, sexuality diverse, homeless

### Theme 2: Healthcare journeys are shaped by decisions weighing up convenience, engagement, perceived effectiveness and affordability

Participants described ambivalence about engaging with the health system. Their ambivalence arose due to the complexity of the health system and the need to repeatedly compromise by weighing-up factors (such as convenience, engagement, perceived effectiveness and affordability), whose importance varied at different points in time. Ambivalence led to delaying healthcare access and foregone care. Participants’ decision-making about when and how to access healthcare was driven by need but also by weighing up any given set of barriers at different times. Navigation became a trade-off about which aspects of service were most important to them at any point: services that are convenient, engaging, effective and affordable.

In deciding to access services, choice was primarily not about finding the most appropriate health service but assessing how the service compared in a trade-off about what was important to the young person. The factors they prioritised varied. Convenient access (flexible, timely access to healthcare services that were easy to get to and open at convenient times) versus engagement (welcoming services with appealing environments with a professional who had the knowledge, skills, attitudes to form a confidential ongoing trusting relationship) was a common trade-off. Services which had convenient drop-in free appointments were viewed positively, but the short appointment times and lack of staff skills in engaging young patients was seen as a deterrent.

*There’s not a whole lot of patient care involved. I’ve been reticent to go back to that medical centre, but I may end up having to because they’re the only place I know in my area that’s open past 6:00.* -Female, 23 years, sexuality diverse, homeless

Effective care was another factor weighed in decisions about access. Professionals who could manage complexity, provide holistic treatment, and helped them understand healthcare issues, treatment and follow-up steps were valued.

*Over the past six months, I’ve seen five individual psychologists. Four of them were dreadful… a lot of it I felt is very pointless and… hasn’t advanced my treatment whatsoever… I spent five months being wrongly medicated… and seeing numerous different people that weren’t advancing my treatment whatsoever. -*Female, 16 years, rural, gender diverse

Costs were prohibitive, hidden and experienced across all levels of the health system. Ability to pay drove choice: cost barriers frequently overrode other types of barriers, leading to foregone care (e.g. dentists were frequently seen as out of reach due to cost and many young people did not access a dentist across the study period).

*I don’t really like and know much about like dentists. Besides, they cost money. The only place I can go for free is at the hospital. And to get a check-up, you know, at a regular dentist, I just assume it would be a lot of money and I don’t have that. Its two years [since I last went to the dentist]… I can’t get in for free, so… it’s too difficult because I don’t have a job or anything.*-Female, 19 years, sexuality diverse, rural, homeless

Although confidentiality was viewed as important, it was commonly understood and expected. Concern about privacy was more pronounced for participants living in rural areas and led to forgone care. Refugee young people expressed confidentiality concerns relating to the use of face-to-face interpreters.

### Theme 3: Marginalised young people perceive and experience multiple forms of discrimination leading to forgone care

Participants perceived and experienced prejudice and discrimination. Discrimination was due to cultural background, Aboriginality, sexuality, gender and age. Many felt misunderstood, judged or not taken seriously. Participants’ experience of discrimination was a major deterrent to care. After negative experiences, even when participants needed care, many would choose to forgo care rather than risk further discrimination.

*We’re just seen as the troublemakers or something in there. I hate hospitals. ... The way they treat me compared to everyone else, it makes me not want to go to hospital. I collapsed the other night and I did not go to hospital, because I hate it. They’re just so rude. They don’t care. -*Female, 18 years, rural, sexuality diverse, homeless

Refugee young people described discrimination based on their cultural background, language fluency and female sexuality (seeking health care for sexual and reproductive health was sometimes met with judgemental attitudes about female sexuality from doctors of their own cultural background resulting in suboptimal care.

*You don’t get taken that seriously… Like everywhere you go, unless you present yourself like someone that can be listened to, you will be passed around like a ball, like over and over again... ‘Cause you have to say things in a certain way for you to actually get the outcome. -*Female, 21 years, refugee

*…so whenever they saw that we are wearing the scarf…they judge* [us]. - Female, 18 years, refugee

Sexuality and/or gender diverse participants often felt misunderstood by health professionals, who lacked understanding about their experiences and needs. Transgender young people experienced specific challenges due to some professionals’ gatekeeping role to access specialist treatment, yet limited understanding of transgender issues. Gender diverse participants also experienced discrimination by service systems, such as failure to acknowledge gender and preferred pronouns or to use interpreters.

[The optometrist] *is best definitely... reason for that is, I’ve been asked when I go in there what my preferred gender is, that was nice.*-Transgender woman, 22 years, sexuality diverse, homeless, rural

Aboriginal participants commonly drew on family advice but felt shame about help-seeking when family and community expected they should be self-sufficient and not need to ask for help. They also appreciated the availability of low-cost services but felt there was stigma about identifying as Aboriginal in order to access them. Community campaigns to address family and community attitudes that reinforce stigma and shame about help-seeking were suggested.

*Sometimes it comes down to... I feel a bit too proud to ask for help*… *or I worry the people will judge me. I’ve got some family that are like, “You know, you don’t need help.” … being part of this family culture as well.*-Female, 24 years, Aboriginal, rural

Participants said they wanted to be treated with respect and valued professionals who were welcoming, caring, non-judgemental and understanding. Welcoming signals – such as rainbow and Aboriginal flags – were seen as positive ways for services to recognise diversity. Some participants suggested youth participation in service design could improve service appeal and relevance.

*I kind of just look at what a doctor’s surgery puts on their own website… If* [the service] *isn’t explicitly trans-friendly or recommended by someone queer and trans-friendly, it’s eliminated. More and more people are coming out as gay and lesbian, transgender... finding a GP for those people* [is] *quite a difficult thing. For me I was like, it’s not worth the hassle. I’ll just live with it. And if something gets bad, I’ll go to hospital. That was how I spent those two years without a GP. Because I thought, hey if it’s that bad, they would send me to hospital, I’ll be fine.*-Trans woman, 23 years, sexuality diverse, homeless

### Theme 4: Multiple marginalisation makes health system navigation more challenging

For many participants access and navigation were difficult due to the lack of relevant services, financial and practical support, and daily life challenges. The barriers varied by age, and level of family support was a significant factor influencing capacity to access and navigate health care. Participants spent periods working hard at attempting to access services and periods of giving up or feeling overwhelmed.

*My life got a little bit insane and I forgot to make a lot of appointments… it can be a bit confusing trying to keep up with them all, because I have to see so many people.*-Female, 23 years, gender and sexuality diverse, rural

Belonging to multiple marginalised groups added complexity to the range of access barriers participants experienced. For example, the varying types of discrimination that young people experienced more strongly was dependent on their marginalised status. Similarly, some marginalised groups experienced varying access barriers more strongly than other groups. For example, homeless young people were more affected by a lack of family support and costs issues, rural young people experienced more issues relating to service availability and confidentiality concerns (relating to visibility accessing services). The effects of multiple barriers were compounded for young people belonging to multiple marginalised groups.

Health care access issues also contributed to young people becoming more marginalised. For gender diverse and rural participants there was an interaction between seeking healthcare and homelessness. Gender diverse participants spoke about previously moving from rural areas to the city to access healthcare but also becoming homeless. One homeless gender diverse participant moved to a rural area to access accommodation but found a lack of appropriate health services.

*It was an issue with the GP not knowing about trans issues. And that was definitely part of the decision to move into a city again, just access to services, because it was quite abysmal being in the middle of nowhere. Yeah, distance wasn’t actually that much of an issue. It was the attitude mostly that was the issue.*-Transgender woman, 22 years sexuality diverse, homeless, rural

Participants felt that professionals under-recognised the impact of marginalisation on their ability to navigate a complex health system. Many did not follow-up referrals due to chaotic lifestyles, not prioritising or recognising the importance, access barriers (e.g. cost, availability), or a lack of support – opportunities to intervene early were lost.

*Finding services has been difficult, remembering to get referrals and then to follow up on them, finding the time to do those things… I do know it’s something I should into because it bothers me, frustrates me a lot… I always tend to lose touch with my mental health professionals right when I need them… The times I could really use someone is when it’s hard to get access.*-Female, 23 years, sexuality diverse, homeless

### Theme 5: The impact of health system complexity and fragmentation may be mitigated by system knowledge and navigation support

Participants commonly described confusion and substantial amounts of time when trying to understand how to access the range of services they required. This varied with the levels of family support they received and also within the context of their developmental stage – those who were younger or less autonomous were less experienced in navigating health care.

Participants described particularly the first visit to a new service as confusing. They were often unclear about if they needed to access health services, and which service would best meet their needs, however this improved across the study period with new experiences of accessing a greater variety of healthcare services. Further, some refugee participants described having additional responsibility for supporting family members’ navigation.

*If you wanna go to a special doctor - you just can type into the network and see… we can help our parents like this or – and because the old people – I know a lot of people can use it – the network and everything, but most of them… they can’t. When they go to the doctor, we can go there and something like this… I search a lot.*-Male, 16 years, refugee

Participants highlighted system inefficiencies and the need to retell their story. Conflicting advice from healthcare professionals added confusion. A lack of engaging and appropriate services led to inefficiencies, as participants looked for alternative more appealing services resulting in missed opportunities and treatment delays.

*The physio is a bit dodge – I didn’t go back to him purely because I didn’t like him… he gave me the creeps… I’m trying to get a GP appointment to get a referral to another physio. I think the biggest thing… is how many people I’ve had to see before I found people that I can work with on my health… some people are more understanding how much my mental health does affect my everyday life and my other health things…*-Female, 23 years, gender and sexuality diverse, rural

Although participants were ambivalent about their health care journey, health care navigation was made easier by understanding the healthcare system, care pathways and how to access low-cost services. They highlighted that this is not currently taught in school but should be.

*More information of how it works. Like bulk billing… that sort of thing… just more information makes the whole process a little less scarier [*sic*]… ‘cause it would probably be the hardest to access it the first time but after having that information, it would probably be easier.*-Female, 20 years, rural, Aboriginal

Participants also appreciated support to navigate systems and helped them find convenient free or low-cost healthcare including professionals and community-based youth workers, refuge workers, refugee liaison workers and Aboriginal health workers. This was particularly evident for homeless participants who lacked family and financial support and found locating welcoming services a challenge.

*A support person to help them go through the process of accessing – just to give them some guidance… and maybe just checking up on them every now and then to… see if that’s going well. If it’s not, they can help guide them on the right way to do that.*-Male, 18 years, homeless

Participants found it difficult to connect with a regular GP, but those who did valued an ongoing trusted connection and support to plans and coordinate care. There was a lack of follow-up by services – for example, many participants who visited an emergency department did not inform their GP or keep their paper-based discharge summary, and this led to healthcare issues not being addressed across time. Transitions between service systems were described as challenging including from paediatric to adult care for participants with a chronic illness.

*A lot of those services stop, [but] it’s not like your health issues just disappear. And it can be hard when you get to that age-out period and all of a sudden you have no support, you’ve got no access to services, and there’s nothing, and you just feel absolutely alone. Then trying to re-navigate everything can be very overwhelming.*-Female, 24 years, Aboriginal, rural

Participants had varying levels of knowledge about after-hours crisis options. While many saw the emergency department as the only available option, some participants used nurse-led telephone helplines to work out the severity of their condition, get advice on self-management, and which service they could access. They also suggested such a service could be run in an online chat format.

*A chart that says either the steps that you can go through or this can be a pathway. There’s too many different ones and it’s all so confusing and so complicated, they’ve made it so much more complicated than it needs to be.*-Female, 18 years, rural, sexuality diverse, homeless

## Discussion

This longitudinal study quantified and described access to, journeys within, and experiences of, the NSW health system over an 18 month period by young people belonging to one or more of five marginalised groups. At baseline, the majority had high levels of mental distress and chronic illness. Just 68% of participants rated their health as ‘excellent’, ‘very good’ or ‘good’; substantially fewer than the general Australian youth population (91.1%, young people aged 15–24, 2014–2015) [22]. Despite having high levels of health need, access and engagement with services by participants fluctuated over time. .

Our initial analysis resulted in descriptive themes that support and extend other models of healthcare access that identify the following dimensions: ‘approachability’; ‘acceptability’; ‘availability and accommodation’; ‘affordability’; and ‘appropriateness’ [23]. In addition, we identified ‘understandability’, ‘convenience’ (services need to be easy to access in addition to being available), and ‘equity’ (health services are non-discriminatory, respectful and welcoming for all young people) to be important for marginalised young people’s healthcare access.

To our knowledge, this is the first longitudinal study from a high income country to explore health system navigation [4]. Overall, we found that marginalised young people are ambivalent about their healthcare journeys and marginalisation makes health system navigation more challenging. The findings indicate that the current health system is not meeting the needs of marginalised young people, but participants provided solutions to address the weaknesses in the health system that could make health system navigation easier.

This is the first study to explore the role of technology in marginalised young people’s healthcare access, engagement, and navigation [4]. Overwhelmingly participants from a range of marginalised groups suggested that technology and social media have an important role in supporting young people’s health literacy, ability to contact and engagement with services. Social media could be utilised to reduce stigma about help-seeking and promote service awareness. Almost all participants had internet access and online information was integrated into their help-seeking decisions [24]. Participants requested that services utilise technology (e.g. SMS, email, online messaging) to facilitate communication with services from the first point of contact through to follow-up – extending previous findings with a rural youth sample [6]. Consistent with a previous Australian study, we found that accessing online mental health support led to some young people seeking face-to-face healthcare [25]. However most participants did not see online services as replacing face-to-face care. Technology solutions are key to implementing navigating solutions for marginalised young people but require systematic evaluation.

We found that young people’s healthcare journeys are shaped by an ongoing process of deciding about healthcare access, weighing up convenience, engagement, effectiveness and affordability. Out of pocket costs and varying governance structures between services (due to different funding, management and operational systems within the Australian health system) as well as variable intake criteria for health services, led to fragmentation of care. Yet in a related study cost was frequently overlooked by health professionals [26]. Our findings suggest that health professionals can play a more active role in facilitating access and supporting navigation by providing better information about cost, service approach, practical advice about attending face-to-face services and online interventions.

The health system can do better to provide welcoming and engaging services that recognise the needs of diverse groups of young people. The use of welcoming signals (such as rainbow and Indigenous flags) by services and including space to record preferred name and pronouns [27, 28] are examples of recognising diversity and guiding interactions. Professional development for all staff in the needs and approaches to working with marginalised young people are warranted [27, 28].

This study is the first from a high-income country to explore health system navigation for rural, refugee and Indigenous youth populations [4]. In addition to the access issues previously identified for rural groups (such as service availability and social proximity [29]), we found that young people living in rural areas had difficulty understanding the full extent of the health system available in other locations. Refugee young people were not only learning about health services [30] and health system for their own navigation, but they also supported family members. Due to confidentiality concerns for refugee young people, telephone interpreters might be preferred for this group. For Aboriginal young people, strategies to address shame may be relevant [31].

This study is the also the first to explore navigation with young people belonging to multiple marginalised groups [4]. We were able to explore the impact of intersectionality - intersecting social categories, that result from belonging to multiple marginalised groups, compounding disadvantage [32]. The way that marginalised young people interacted with services and navigated the health system was affected by multiple disadvantage. Despite frequent contact with health services, lack of engagement, negative experiences or system complexity led to ambivalence, foregone care and inefficiency. Belonging to more than one marginalised group compounded these difficulties. Without recoginsing intersectionality and its impacts, health services and systems may therefore contribute to inequity for marginalised young people. Many participants felt professionals lacked an understanding of how multiple disadvantage impacted on their ability to follow through on health care, indicating a need for professionals’ training and youth participation in service design. Currently, there is a lack of youth participation in service design or review, the promotion of which should facilitate access.

Professionals can do more to support marginalised young people’s navigation from the point of entry to better managing the transition from adolescence to adulthood in the health system. Although the health system is complex and fragmented, system knowledge and navigation support are helpful. Professionals should look beyond single encounters to consider young people’s holistic health and wellbeing needs by playing a more significant advocacy role so marginalised young people can access affordable services (e.g. by negotiating free access to referred services) [26]. Entry points into the health system (e.g. Emergency Departments) can help young people identify and connect with appropriate care – but proactive follow-up support is needed.

Although data on healthcare access showed participants used a wide variety of healthcare service types across the health system, GPs were the most frequently accessed health professionals. Trusted GPs played a central role in planning participants’ health system navigation when they considered participants’ health holistically and understood access barriers. However many GPs find working with adolescents challenging, particularly as systems can be restrictive (e.g. most general practice consultations are time-limited due to funding structures, so that long or extended appointments are unusual) [33]. Other community-based workers, (such as refuge workers), supported health system navigation, indicating the significant role of non-health professionals [34].

The findings also suggest increasing system knowledge can empower marginalised young people to appropriately meet their health needs. Participants wanted to learn more about the health system and its navigation, both in school and online. Empowering young people to understand the health system and access care has the potential for health conditions being managed earlier in the illness trajectory.

In a related study, health professionals identified the need for health system reform to better support young people, particluuarly those who are marginalised [26]. Participants views were consistent with the views of professionals, that health services need to embrace technology, better engage diverse groups and support young peoples’ health system navigation.

Youth-friendly service models [35] could move beyond making single health services accessible and considering their place within the whole health system navigation. New approaches would support navigation by facilitating understanding of the health system, help-seeking decision-making, reaching services and follow-up. Youth-friendly models of care could then proactively support young people’s dynamic journeys through the health system [3]. Although challenging, reducing system complexity would make health system navigation easier – flexible service structures and integrated care may be opportunities to achieve this.

Policy implications from our study include a focus on marginalisation and intersectionality on the one hand, and service reorientation to incorporate health system navigation on the other, integrating technology into service planning and delivery. In NSW, the youth health policy (which this study informed) has a focus on engaging vulnerable young people, the use of technology, developing care pathways, and supporting young people to access and navigate health services. The policy, however, also acknowledges that services and professionals need training and support, for example to optimise the potential of technology [36].

Our study has several limitations. Participants were self-selected with high levels of health need. While the sample size was small, retention of over 85% of the sample and conducting up to four interviews across a year helped deepen the researchers’ understanding. Some refugee participants with English language difficulties chose not to have interpreters present, and one chose not to be recorded. Our findings are limited by the number and choice of marginalised groups. However, as the first study to include multiple marginalised groups, we found intersectionality theory a useful lens to understand multiple disadvantage.

## Conclusion

This study is the first to explore marginalised young people’s access and navigation across time. Participants offered salient solutions to address the weaknesses in the system and services including integration of technology and social media into processes that facilitate access and navigation, recognising and respecting diversity through providing welcoming and respectful services, and improving health literacy to better equip marginalised young people in their decisions about accessing health care. Further, to achieve equity, and universal health coverage, system knowledge, advocacy (particularly in relation to cost) and active navigation support for those who are marginalised and most disadvantaged is needed. Without this support, many marginalised young people fall through the gaps of a system that is intended to care for them.

Exploring intersectionality was not the main purpose of this study but was an emergent finding. Belonging to multiple marginalised groups, can make health system navigation even more complex. Intersectionality theory can bring additional explanatory power to understanding why some young people experience poorer health outcomes. Intersectionality recognises that individuals can have multiple social identities which impact on health equalities. By recognising the additional needs of marginalised young people and using an intersectional lens, health professionals can provide non-judgmental and respectful services that promote and recognise diversity.

## Additional file

Additional file 1: sample interview questions. Marginalised young people’s health system navigation: a longitudinal qualitative study: sample interview questions. This table provides sample interview questions used in the interviews with marginalised young people across the longitudinal study. (DOCX 17 kb)

## Abbreviations

GPs: General Practitioners
K10: Kessler-10
NSW: New South Wales
WHO-5: World Health Organisation Wellbeing Index
