# Supplementary material for: Health care equity and access for marginalised young people: a longitudinal qualitative study exploring health system navigation in Australia
Source: Int J Equity Health. 2019 Mar 4;18:41. doi: 10.1186/s12939-019-0941-2 (PMC6399978; doi:10.1186/s12939-019-0941-2)
Supplement: Supplementary file 1 — sample interview questions. Marginalised young people’s health system navigation: a longitudinal qualitative study: sample interview questions. This table provides sample interview questions used in the interviews with marginalised young people across the longitudinal study. (DOCX 17 kb) [file 12939_2019_941_MOESM1_ESM.docx]

**Marginalised young people’s health system navigation: a longitudinal qualitative study: sample interview questions**

| **Interview 1**  **Health information**  Where do you go looking for information about health?  How do you find out about health services?  How do you decide if you need to access healthcare?  Do you get advice from others to help you decide whether to access healthcare?  Does the internet play any role in choosing health services? How?  What different kinds of health services have you been to over the past 6 months?  **Exploring service encounters**  Thinking about before you went to the service, how did you decide what to do and find out where to go?  Did you find out about it yourself or did someone else suggest it to you? Did you look online?  How did you decide if you needed to attend or not?  Could you describe your experience at the service?  How did your contact with the service help with understanding about your health?  Did you have a support person with you (like a parent or other person)?  If you are comfortable to tell us, what were the recommendations or ‘management plans’ that came from your contact with the service? Please feel free only to give general comments, you don’t need to tell us specific diagnoses or treatment.  Were you involved in planning or deciding about your management plan?  What did you think of the management plan? Did you feel able to act on them?  Were there any things that made it difficult to access the service?  Can you describe any practical issues with accessing the service, e.g. cost, transport, opening hours?  Were there any other issues, for example did any worries get in the way of you attending the service?  Did the service communicate with others – for example your parents, carer, school, or other health professionals?  Can you describe any issues or concerns you had regarding confidentiality or privacy when you had contact with the service?  Were there any other services you’ve been to in the last 6 months that you’d like to talk about?  When you think about all the different health services you have been to over past 6 months, how do you find the experiences of going between different services?  In that past 6 months, were there health issues that you thought about accessing help for but didn’t?  Did anyone suggest that you access a health service – was that helpful?  How does your family or support people influence if you access a health service?  What would you advise other young people in your situation about accessing health services?  **Understanding the health system**  As a researcher we see all the health services together make up the health system. What do you consider makes up ‘the health system’, for example what different kinds of services would you include? [*easy English version:* What other different kinds of health services do you know?]  What do you think it’s like for other young people to access the services they need?  What would make it easier for young people to access health services?  What is your overall impression of health services based on your own experiences or understanding?  How would you like to improve the health system/health services in NSW for young people?  **Interview 2**  **Experiences in the past 3-4 months**  Have you experienced any health concerns since we last spoke 3-4 months ago?  Did you explore getting support? How did you go about deciding where to go and if you needed healthcare?  Did you attend or access a service in the last 3 months?  How did you choose that service, rather than another one?  What was your experience like?  Did you need to go to multiple services? What was it like finding your way around services?  Did anyone help you find where you needed to go?  Did you use technology to help you find your way?  Were there any health issue you thought you’d like to get help for but didn’t for whatever reason?  What made it hard or got in the way?  Did you have any appointments that you didn’t end up attending?  What made it hard or got in the way?  What would make it easier to access the services you need in future?  What is your view about using online services? What do you see as the benefits of online services? What do you see as the challenges?  **Explore System Inefficiency**  Explore this in more detail if it appears to emerge. E.g. if they say they went to two different GPs, or a GP and ED (or whatever) ask whether they found the services they got were similar/ complementary/ repetitive/ (whatever word/s seem appropriate!) - perhaps by probing into whether the professional/s they saw explained things well, or whether they visited a different service the second time because they didn't feel they got what they needed from the first... etc  **Marginalised status**  As a young person growing up (in a rural area/as a young Aboriginal person/being LGBT /living in foster care/living in a refuge/ from a refugee/vulnerable migrant background, how has that influenced your access to health services?  What are the benefits of accessing health services?  What are the challenges?  Can you describe what it’s like to find your way around health services?  **Further exploration of KEY BARRIERS**  **COST**  Are there any services you’d like to access but don’t go to because of cost?  What is the effect of not going to those services?  If the services were free how would that make a difference?  (If over 15) Do you have your own Medicare card or are you on your family card?  How does having your own card make a difference to you?  Do you have to pay a fee when you see the GP? How much do you pay? Is that a lot of money to you?  (If bulk billed) How did you know you would be bulk billed?  Is the costs of going to the dentist a problem for you?  **OPENING HOURS**  What kinds of services are available after usual business hours?  In what ways do the current opening hours affect your ability to access services?  What hours would you prefer?  How would that make a difference?  **EMBARASSMENT**  What makes you feel less embarrassed?  How does this make a difference?  **JUDGEMENT**  What makes you feel less judged?  How does this make a difference?  **System Navigation**  Do you support others to access health services? Have you ever supported a friend to visit a service? Do you support your family to access health services?  What would be the best was for young people to learn about how to navigate the health system?  **Big picture questions**  What would you advise other young people in your situation about accessing health services?  What would make it easier for young people to access health services when they need them?  **Interview 3**  As in the other interviews we are keen to find out any experiences of accessing health services…  **Experiences in the past 3-4 months**  [Begin with same questions as in Interview 2]  **System Inefficiency**  [Begin with same questions as in Interview 2]  [Explore when they describe fragmentation and chaotic services but don't express frustration (e.g. 'The description of how you had to go from xx service to yy service/ had to wait xx for a service (etc) sounds like what we might call inefficient or frustrating - what are your thoughts about how it all worked for you'?)]  **Unstated health needs and openness to explore these with a health practitioner**  What about not accessing services that you probably needed? Did you ever think “I really should find some help” or “I know I’m supposed to go to that service…” but didn’t? What happened?  **Holistic approach to assessment**  In other research studies young people have said that, while they are not always comfortable to seek help or ask about health problems, they are ok about being given information or being asked about those things by a doctor. Some examples of health issues might be sexual health or mental health.  How do you feel about a health practitioner asking about those things, even if you hadn’t gone to the service for that reason? How would that make a difference?  Do you think you normally have enough time in your session with a health practitioner to cover all those things that might be relevant to you?  **Language**  What do you think about the language that health professionals use?  What can doctors do or say to make things clear to reduce misunderstandings?  **Stigma/Shame**  Can you help me understand why young people may feel awkward or embarrassed about contacting or going to a health service?  Do you find you have a different attitude to your parents when it comes to shame/stigma/embarrassment about accessing healthcare?  What could help reduce the stigma or experience of shame for young people?  What are the best ways to help young people feel less embarrassed about contacting a service?  How would that make a difference?  **Discrimination**  What make health services welcoming for different kinds of young people?  Have you ever felt a service wasn’t welcoming?  What you ever felt discriminated against or treated in a disrespectful way?  How could a service be promoted that would encourage access by young people from diverse groups?  How would that make a difference?  **Ideal experience**  Can you describe for me an 'ideal way for getting a health need looked after, starting from the first time you experience it until you feel it's been completely taken care of and you feel fine about it?  **Interview 4**  **Experiences in the past 3-4 months**  [Begin with same questions as in Interview 2]  **Health system navigation**  What would have made it easier to find your way around health services?  How would that make a difference?  How do you use technology to help you navigate your way around health services?  Do you have any thoughts about confidentiality and sharing your information online?  How could technology be better used to help young people navigate the health system?  Do you have other ideas about how it can be made easier for young people to find the services they need?  We’ve been talking for a while about your experience of accessing healthcare and navigating health system. What have you learnt through reflecting on your experience?  Do you have any recommendations about how to make the health system easier to navigate for young people? |
| --- |
